# Early Discharge and Patient-Initiated Follow-Up in Hand Surgery: A New Norm Following Simple Hand Surgery?

**DOI:** 10.7759/cureus.52493

**Published:** 2024-01-18

**Authors:** Haneen Abed, Deepak Samson, Michael David

**Affiliations:** 1 Plastic Surgery, University Hospitals Coventry and Warwickshire, Coventry, GBR; 2 Orthopaedic Surgery, University Hospitals Coventry and Warwickshire, Coventry, GBR; 3 Orthopaedic Surgery, Univeristy Hospitals Coventry and Warwickshire, Coventry , GBR

**Keywords:** plastic and reconstructive surgery, patient follow-up, safety patient, patient-education tool, orthopaedic hand surgery

## Abstract

Aims: The demand for elective hand surgery has increased substantially over the last 10 years. With COVID-19 providing an added challenge of restrictions on face-to-face consultations, already overflowing follow-up clinics will be stressed further. Our aim is to assess the viability, effectiveness, and safety of an early discharge directly from the operating theatre following common hand surgery procedures with the safety net of open-access follow-up.

Methods: All eligible patients undergoing open-hand surgery under local anaesthesia between February 2019 and December 2020 were offered early discharge. Informed consent was obtained in the clinic, and they were counselled on rehabilitation immediately prior to surgery. Patients were given a custom-made "open-access business card" with clear post-operative instructions and hand exercises, along with information on how to get in touch to request clinic follow-up. A review was completed at a minimum of two months following surgery. Administrative support staff were briefed beforehand to minimise any delays in follow-up requests and either book patients who requested follow-up into a routine elective clinic or utilise ring-fenced emergency elective clinic slots depending on the patient's individual requirements.

Results: A total of 105 patients were included in this study, with an average age of 60 years. The average interval between surgery and review was 20 weeks. Eighty-nine patients had a successful early discharge, with 16 patients requesting clinic follow-up. The average time to follow up in the clinic was 35 weeks (range: four to 84 weeks). There were no complications that we were made aware of, and the most common reason for returning to the clinic was a new complaint, unrelated to the surgery.

Conclusions: Although virtual follow-up is now well established in both the fracture and elective clinic settings, early discharge is largely uncharted water. Our pilot demonstrates that early discharge and patient-initiated follow-up for common elective hand surgical procedures under local anaesthesia are efficient, safe, and viable.

## Introduction

The demand for elective hand surgery has increased substantially over the last decade, with increased pressure for both theatre space as well as outpatient services. The added pressures from the impact of COVID-19 have meant that there is a significant catch-up to be made on elective work, with even less clinic availability due to face-to-face restrictions. Changes in the National Health Service (NHS) [1] have involved the redesign of service provision and the application of new policies, owing to financial needs to improve cost efficiency.

In the NHS, between 2019 and 2020, there were over 124.9 million outpatient appointments; this is an increase of 1.3% from the previous year and 66.9% from 10 years ago [2]. Of these, there were 96.4 million outpatient attendees, with the remainder either cancelled by the hospital or patient or where the patient “did not attend" (DNA) [1]. Comparing data from 2009-10 with 2019-20, DNAs have increased by 15%, whereas hospital and patient cancellations have increased by 124% and 92.7%, respectively. As part of the NHS redesign strategy [3], the ‘High Impact Changes’ document was created, which emphasises the need to avoid unnecessary follow-up appointments and encourages a one-stop streamlining approach for outpatient clinics. The University of Manchester provided a report with similar recommendations [4], advocating that routine follow-up appointments could be replaced with no follow-up, patient-controlled follow-up, or primary care follow-up.

Although the concept of virtual follow-up is now well established in both the fracture and elective clinic settings, the concept of early discharge and patient-initiated follow-up (PIFU) is largely unchartered water in hand surgery. We investigated this further through a service evaluation within elective hand surgery. The aim of this study was to assess the viability, effectiveness, and safety of an early discharge following common hand surgery procedures, with the safety net of open access follow-up. We hypothesised that patients could be discharged directly from the operating room following a simple day-case procedure performed under local anaesthesia.

This article was previously presented as a meeting abstract at the British Society for Surgery of the Hand (BSSH) Autumn Scientific Meeting on September 10, 2021.

## Materials and methods

Following local approval for this prospective study, all patients undergoing simple open surgery under local anaesthesia between February 2019 and December 2020, under the care of a single consultant hand surgeon at University Hospitals Coventry and Warwickshire, Coventry, GBR, a university hospital and major trauma centre, were selected. Inclusion criteria included language compatibility, access to a telephone, and suitable social factors with easy access to transport to ensure patient safety was maintained. The hand procedures included in this study were: carpal tunnel decompression, trigger finger release, and steroid injection into the first carpometacarpal joint. Patients with a combination of procedures, multiple trigger finger releases, or endoscopic bilateral carpal tunnel releases were excluded from the study.

**Figure 1:**
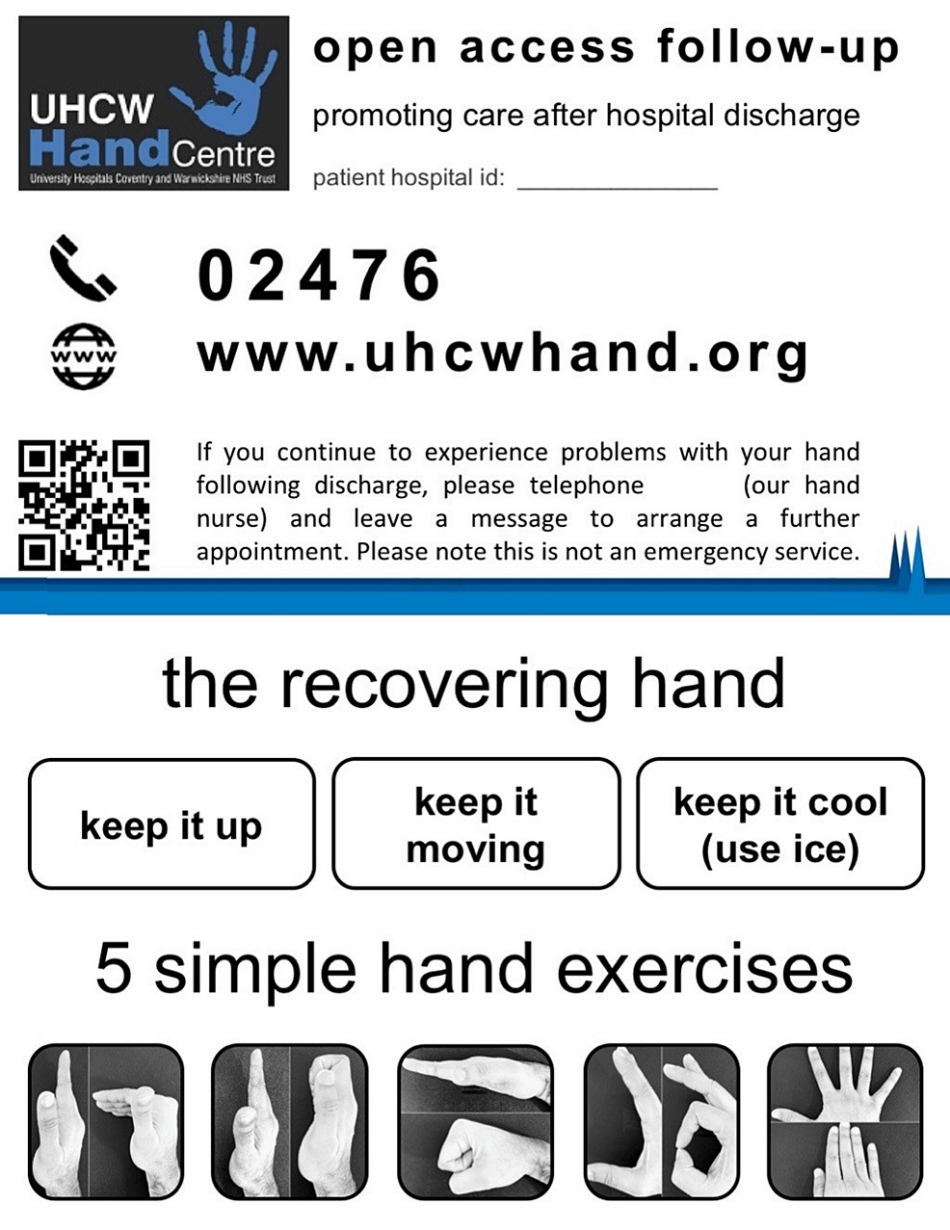
Open-access business cards provided to patients upon patient-initiated follow-up (PIFU)

All patients were reviewed by the surgeon in the clinic prior to their operation, where informed consent was obtained for the procedure, eligibility was confirmed, and patients were counselled on the PIFU processes. Patients were counselled on rehabilitation immediately prior to surgery and provided with a custom-made "open-access business card" with simple post-operative instructions and hand exercises, along with information on how to get in contact to request clinic follow-up (Figure 1).

A combination of both written and visual instructions for hand exercises was provided on the business card to help with understanding. Business cards were only provided in the English language in this study. A website link to the hospital's hand surgery webpage was also included on the business card, which allowed patients to access patient information leaflets as well as useful contacts from home. These cards were designed with a multidisciplinary approach, including contributions from consultant hand surgeons and hand therapists.

No routine outpatient clinic follow-up was arranged with the surgeon post-operatively, and instead, all patients received an early discharge directly from the operating theatre. Any wound review, change of dressing, or suture removal was arranged with the patient’s general practice or district nurse.

Administrative support staff were briefed beforehand to minimise any delays in follow-up requests. They were advised to either book patients who requested follow-up back into a routine elective clinic slot or utilise ring-fenced urgent elective clinic slots depending on their individual requirements and availability. Patients who contacted the department expressing a desire to be reviewed by the operating surgeon were seen within two weeks. 

Data were collected on the patients' demographics, procedure undertaken, operation date, and whether a review was requested by the patient. If a follow-up was arranged, the date of the follow-up and the indication for follow-up were recorded. We prospectively reviewed the data at two intervals: initially at a minimum of two months, and then again at a minimum of six months. Two months was deemed a reasonable period to highlight if there was a post-operative concern or complication. The six-month interval was considered the second review point whereby any acute or chronic post-operative concerns may have been raised.

All data were collected electronically using the hospital's electronic records system. As part of the study, patients were asked to give feedback on their experience of the early discharge process, what they felt about PIFU, and what they thought about the open-access business card provided. An anonymous survey consisting of white space answers as well as five single-answer questions was distributed a year after the completion of the study.

Departmental management and local ethical approval were obtained for this project from the University Hospitals Coventry and Warwickshire Local Ethics Committee, Coventry, GBR (approval number: 1584).

## Results

A total of 108 patients were recruited for this study. Two patients failed to meet the inclusion criteria, and one patient declined PIFU. The average age of the cohort was 60 years (range: 19-94 years). The patients included in the study had one of three simple procedures performed under local anaesthesia: a total of 83 patients had a carpal tunnel decompression procedure, nine patients had a trigger finger release, and 13 patients had a steroid injection into a joint.

Of the total 105 patients, 85% (89 patients) had a successful early discharge and did not require any follow-up. The remainder of the patients (n = 16) requested clinic follow-up. The average time to request follow-up was 35 weeks (range: four to 84 weeks), and the most common indication was for a new or different problem, unrelated to their surgery. Four patients used the open-access service to request an operation on the contralateral side; these were mainly patients who had an open carpal tunnel decompression procedure. Further, four patients requested follow-up to discuss a new problem: triggering of a digit, De Quervain’s tenosynovitis symptoms, and ulnar nerve symptoms. One patient was booked in error. The remaining seven of the 16 patients who requested a follow-up appointment wished to discuss a problem related to the original surgery (Table 1).

**Table 1:** Reasons for follow-up requests following discharge

The reasons for 16 patients requesting follow-up	
Request for an operation on the other side	4
New problem	4 (2 for pillar pain, 1 for scarring, 1 for stitch reaction)
Related problem	7
Clerical error	1

The most common reason was scar pain or pillar pain, and all were managed successfully with hand therapy, analgesia, or reassurance.

For those patients who made an appointment using the open-access cards, feedback was obtained on how they felt their experiences were with PIFU, with no formal follow-up being arranged. All participants in the study were contacted a year after they had their surgical intervention. An anonymous survey was distributed, and follow-up telephone calls were also made. A total of 22 patients provided feedback on their experiences. The overall feedback was positive, with many patients finding it an easy process with an appointment made very quickly. All patients found the open-access cards a good idea, and in fact, one patient felt that an earlier appointment to discuss similar symptoms on the other side would not have provided any additional benefit. Although this early discharge and patient-led follow-up had originally been initiated prior to the current climate, the COVID-19 pandemic has emphasised the need for a reduction of face-to-face interactions as well as ensuring that safety is maintained. This was reflected in positive feedback from the patients, staff, and hospital management. A sample of some anonymous feedback obtained from patients is as follows:

“I was a little nervous about not being followed-up initially, but I had no problems and the contact card reassured me”

“With COVID-19, I only wanted to come to hospital if I needed to and this felt like a safe alternative”

“Really easy process to get into contact for advice with the card, and an appointment was made quickly” 

## Discussion

The surgical outpatient clinic is the first point of contact between patients and their surgeons, where they are assessed, investigated, and treatment plans advised. Following surgical intervention, follow-up practice varies amongst surgeons, departments, and hospitals. It is deemed standard practice to follow up on all patients post-operatively at a follow-up clinic; however, the value of this practice has been questioned [5]. Within elective hand surgery, while some surgeons do not follow up on standard procedures such as carpal tunnel decompressions and trigger finger releases, others advocate routine follow-up, citing the need to monitor progress, patient preference, and general practitioner concerns about increased workload [6]. To our knowledge, there has been no definitive evidence to show that a no-follow-up policy is safe and acceptable for patients undergoing hand surgery. With this in mind, we conducted an evaluation of service within simple, local anaesthetic elective hand surgery.

Although there has not been much evidence of PIFU, the concept of virtual follow-up is being implemented widely within various surgical specialities. A randomised controlled trial undertaken by Healy et al. [7] assessed whether virtual outpatient clinics were an acceptable alternative to face-to-face outpatient clinic attendance for a broad range of surgical patients. They looked at a total of 209 patients and found the majority of patients discharged from a surgical service could be better followed up by a virtual clinic, with a significant proportion of patients reporting a preference for and greater satisfaction with such a service. What is interesting about these results is that a similar telephone consultation to that used in our study was used to evaluate the overall experience of the PIFU process and positive feedback was obtained. A step beyond the virtual clinic follow-up would now be undertaking a randomised controlled trial into the use of PIFU compared with follow-up. To date, this is something that has not been undertaken in the literature.

Over the years, the doctor-patient relationship has evolved, with patients having a much greater say in their healthcare. Within surgical specialities, such as hand surgery, outcomes can often be dependent on patients’ self-care. In 2004, the government recommended a “no routine follow-up policy” to improve NHS efficiency [3]. The policy was aimed at reducing the inconvenience to patients of multiple trips to the hospital. Although this guidance has been suggested, to our knowledge, there has been no definitive evidence to show that no follow-up policy is safe and acceptable to patients, or indeed, any evidence to suggest that a mandatory follow-up policy is necessary. In order to address the variance in clinical practice among surgeons and to acknowledge both patient and clinician concerns, we undertook a service evaluation in simple elective hand surgery that has demonstrated that PIFU is a safe and acceptable alternative to formal follow-up. Although this was undertaken on a smaller scale, it demonstrates the start of a different way of managing post-operative care.

Overall, 85% of patients reviewed were discharged successfully and safely, directly from the operating theatre after simple, local anaesthetic day-case hand surgery. Of those requesting follow-up, the associated workload was managed through ring-fenced appointment slots reserved for these patients. For this process to be effective and to ensure adequate engagement with the process, a significant amount of time will need to be spent pre-operatively on communication. Patients often rely on information from perioperative counselling and written discharge instructions [8]. Inadequate or unclear information, as well as unanticipated issues, can lead to additional and often unnecessary utilisation of healthcare resources [9]. Although this can be seen to utilise more clinical contact time pre-operatively, it is arguable that this would overall reduce the clinic time and therefore outpatient clinic burden that can be utilised for new patient referrals or any other clinical duties required by the surgeon in question.

Sixteen patients requested follow-up through this PIFU process. The indications for follow-up were varied. A total of four patients utilised the PIFU card to discuss carpal tunnel syndrome symptoms on the other side. Having investigated this further, all four patients either complained of mild symptoms on the initial clinic assessment, and therefore a carpal tunnel decompression earlier was not deemed appropriate, or had negative nerve conduction studies. As part of this PIFU process, patients who were predicted to require further hand surgery or interventions were excluded from this study. The four patients who did request the procedure on the contralateral side, interestingly, all made contact beyond the standard follow-up period of four to six weeks [10], as they did not feel ready to consider surgery sooner.

Of the remaining 16 patients requesting follow-up, four contacted their surgeon with an unrelated presenting complaint, seven for a problem related to their surgery, and one was a clerical error. The new problem was either due to pillar pain, scarring, or a stitch reaction, all commonly associated problems with these simple elective hand surgeries [11]. The new problems included dressing advice or addressing general queries about levels of activity. Some of these follow-up requests were factors that could be addressed in a virtual format with telephone consultation rather than face-to-face, and some of these requests were from patients who would benefit from the reassurance that does not require a consultant-led clinic but rather a healthcare professional such as a specialist nurse practitioner. Addressing these factors could, in fact, reduce the follow-up requests even further. As part of the pre-operative planning, patients are thoroughly counselled on what to expect after the procedure. Patient information leaflets are provided with good patient-led post-operative care. Ring-fenced appointment slots were consultant-led clinic appointments reserved for those who requested them. Nurse-led specialist appointments providing telephone advice and reassurance could address these queries in the future.

As part of the study, patients were contacted via telephone consultation to obtain feedback on three major aspects of this service evaluation: 1) experience of the early discharge process; 2) feelings towards PIFU; and 3) feedback on the open-access business card. A quarter of the patients provided feedback, with an overall positive response. Many patients felt that they would prefer not to attend appointments if not required, particularly professionals requiring them to take time out of work to attend and parents with younger children. This sentiment was enhanced during the COVID-19 pandemic when anxieties were heightened at the prospect of entering the hospital for face-to-face contact [12]. Nearly all patients gave positive feedback with regard to taking ownership of their healthcare, but there were a small number who considered this a source of anxiety. The anxieties were often associated with ensuring the patient's recovery was as expected [13], as well as the loss of specialist contact for reassurance. All patient feedback was complimentary of the open-access business card, both from an aesthetic perspective and also because it was a small form that could easily fit into wallets or purses.

Our service evaluation had some limitations that should be considered. Our study only used a single surgeon's practice, which may limit generalisability, so further research evaluating the use of PIFU with multiple surgeons in multiple centres is needed. We tried to keep dedicated ring-fenced slots available for the purpose of this study; however, when thinking about the long-term maintenance of the PIFU process, it is arguable that these ring-fenced slots would potentially be wasted clinic time if not utilised. The counterargument is that without the ring-fenced slots, there is a potential delay in patients getting a clinic appointment upon request. This is offset by the number of clinic slots, both virtual and face-to-face, that would be freed up, with an 85% success rate demonstrated in our study with early discharge following surgery. Furthermore, a potential solution to this would be to audit the average number of utilised vs. unutilised clinic appointments and ring-fence slots based on departmental experiences. Demands and needs can be altered by regular monitoring.

The study was limited to simple hand surgery procedures, covering only unilateral carpal tunnel decompression, trigger finger release, and steroid injection into the first carpometacarpal joint. Perhaps it could be extended to other common, smaller elective procedures performed under regional anaesthesia, for example, cubital tunnel decompression, ganglion excision, and segmental fasciectomy, although many of these options would only be possible provided there was a reliable and robust hand therapy service available post-operatively [14].

The PIFU process requires patients involved to be able to understand the process, have access to telephone devices, and communicate if any concerns arise. This was highlighted early on in this project, particularly with a large number of patients being in the older age category. Efforts were made throughout this project to counter this by ensuring, where necessary, a next of kin or carer was present and/or involved in the discussions, with some acting as power of attorney and engaging in the process.

One limitation from a clinician’s perspective is limited exposure to post-operative successes. A large proportion of job satisfaction is associated with improving patients’ symptoms or alleviating discomfort [15]. This feedback is lost when no follow-up is made, as is the reassurance that operative techniques are suitable. One could argue that no news is in fact good news, and this indirectly provides feedback that the surgery was most likely satisfactory. Contrastingly, it is important to take into consideration that just because a patient has not reached out to you for a review, that does not mean they have not sought a review with another clinician.

## Conclusions

Early discharge of patients directly from the operating theatre can be safe and successful following simple, local anaesthesia day-case elective hand surgery. Patient-initiated follow-up is a relatively new concept within surgery, and patients have embraced the concept of taking charge of their healthcare. The reduction in outpatient clinic utilisation for these follow-up appointments that are no longer required frees up slots for other purposes. These valuable outpatient resources can help tackle waiting lists and the ever-growing needs of the NHS. With the substantial impact of the COVID-19 pandemic, not only does this minimise face-to-face contact to help reduce transmission, but these services will also prove worthy in reducing the elective backlog and supporting the healthcare needs of patients. There is also scope to utilise PIFU in other surgical or medical specialities, particularly where minor procedures are performed, and therefore this is an area for further research.
